# Detection of ESR1 mutations in circulating cell-free DNA from patients with metastatic breast cancer treated with palbociclib and letrozole

**DOI:** 10.18632/oncotarget.11383

**Published:** 2016-08-19

**Authors:** Rekha Gyanchandani, Karthik J. Kota, Amruth R. Jonnalagadda, Tanya Minteer, Beth A. Knapick, Steffi Oesterreich, Adam M. Brufsky, Adrian V. Lee, Shannon L. Puhalla

**Affiliations:** ^1^ Women's Cancer Research Center, Magee-Womens Research Institute, Pittsburgh, PA, USA; ^2^ Department of Pharmacology and Chemical Biology, University of Pittsburgh, Pittsburgh, PA, USA; ^3^ University of Pittsburgh Medical Center (UPMC) Presbyterian, University of Pittsburgh, Pittsburgh, PA, USA; ^4^ Department of Medicine, Division of Hematology/Oncology, University of Pittsburgh Cancer Institute and UPMC Cancer Center, Pittsburgh, PA, USA

**Keywords:** ESR1 mutations, metastatic breast cancer, circulating cell-free DNA, aromatase inhibitor, cdk4/6 inhibitor

## Abstract

ESR1 mutations are frequently acquired in hormone-resistant metastatic breast cancer (MBC). CDK4/6 inhibition along with endocrine therapy is a promising strategy in hormone receptor-positive MBC. However, the incidence and impact of ESR1 mutations on clinical outcome in patients treated with CDK4/6 inhibitors have not been defined. In this study, we evaluated the frequency of ESR1 mutations in cfDNA from 16 patients with MBC undergoing palbociclib and letrozole therapy. Four common ESR1 mutations (D538G, Y537C, Y537N, and Y537S) were analyzed in serial blood draws using ddPCR. Mutation rate was 31.3% (5/16) (n=3; *de novo*, n=2; acquired). D538G was the most frequent mutation (n=3), followed by Y537N and Y537S (n=2). One patient showed multiple ESR1 mutations. Mutations were enriched during therapy. Progression-free survival (PFS) and overall survival (OS) were similar in patients with and without mutation detected at any given time during treatment. However, PFS was significantly shorter in patients with ESR1 mutation at initial blood draw (3.3 versus 9.0 months, P-value=0.038). In conclusion, ESR1 mutation prevalence is consistent with recent studies in hormone-refractory breast cancer. Further, treatment with palbociclib and letrozole does not prevent selection of ESR1 mutations in later lines of therapy. Larger studies are warranted to validate these findings.

## INTRODUCTION

Estrogen receptor alpha (ERα) is expressed in approximately 70% of all breast cancers and endocrine therapy represents a major treatment modality in ERα-positive disease. Tamoxifen, the selective ER modulator (SERM), and aromatase inhibitors have been the standard of care for many years, significantly reducing recurrence rates in early-stage breast cancer [1, 2]. In the last decade, there have been tremendous advances in endocrine therapy with the development of third-generation AIs (anastrozole, letrozole, and exemestane) [3]. These agents are approved as first-line hormonal therapy particularly in postmenopausal women with metastatic breast cancer and have contributed to the improved survival in MBC [4, 5]. Despite these advances, the presence of *de novo* or acquired resistance to endocrine therapies remains a major clinical challenge. Several mechanisms of resistance have been proposed including downregulation of ER expression, cross-talk with growth factor signaling pathways (phosphatidylinositol 3-kinase (PI3K)/protein kinase B (Akt)/mammalian target of rapamycin (mTOR) pathway or the mitogen-activated protein kinase (MAPK) pathway), and cyclin D1 overexpression [6, 7]. In addition, genomic alterations in many oncogenic genes have been identified in ER-positive advanced breast cancers such as PIK3CA mutations, FGFR1 and CCND1 gene amplifications, and more recently ESR1 mutations [8–15]. These signaling molecules provide novel targets to develop more effective therapies to overcome or delay endocrine resistance.

Cyclin D1 and cyclin-dependent kinase 4/6 (CDK4/6) complex pathway regulates cell cycle progression from G1-phase to S-phase by phosphorylation and inactivation of the retinoblastoma protein (Rb) [16]. Cyclin D1 gene amplifications and/or protein overexpression has been shown to predict poor clinical outcome in a subset of ER-positive breast cancers [17]. Also, in preclinical models of antiestrogen resistance, CDK4/6 inhibition has shown to promote Rb-mediated transcriptional repression and decrease in cellular proliferation [18]. Hence, targeting cyclin D1-CDK 4/6 pathway in the setting of endocrine resistance has gained recent interest for improving the efficacy of existing therapies. Palbociclib (Ibrance, Pfizer) is an oral, reversible, and highly selective small molecule inhibitor of CDK4 and CDK6 [19]. A phase II study (PALOMA-1/TRIO-18) in patients with newly diagnosed ER-positive, HER2-negative advanced breast cancer demonstrated significantly longer PFS (20.2 versus 10.2 months) with palbociclib and letrozole treatment compared to letrozole alone [20]. These promising results led to the FDA approval of palbociclib for use in this setting. Similarly, the phase III clinical trial (PALOMA-3) in patients with HR+ MBC who progressed on prior endocrine therapy showed that palbociclib combined with fulvestrant, a selective ER degrader (SERD), resulted in longer PFS than fulvestrant alone (9.2 versus 3.8 months) [21].

More recently, ESR1 mutations have emerged as another mechanism of resistance to endocrine therapy [10–15]. These mutations cluster in the ligand-binding domain (LBD) of the receptor that result in ligand-independent ER activity. ESR1 mutations are relatively rare in newly diagnosed, treatment naïve breast cancer (less than 7% mutation rates in primary tumor), but appear to be frequently acquired in hormone-resistant MBC (15% - 55%). We have previously reported sensitive detection of ESR1 mutations using droplet-digital PCR (ddPCR) in 7% (3/43) primary ER-positive breast cancers, and in 24% (7/29) cfDNA samples collected from patients with recurrent disease [14]. Our data also suggested that longitudinal tracking of the ESR1 mutations may be predictive for development of resistant disease, an area receiving growing attention. In a recent study, ESR1 mutations were found exclusively in ER-positive breast cancer patients previously exposed to AI [22]. Further, patients with ESR1 mutations were reported to have a substantially shorter PFS on subsequent AI-based therapy. Hence, ESR1 mutations may help guide treatment selection of novel targeted therapies for future management of endocrine resistance. The incidence and impact of ESR1 mutations on clinical outcome in patients treated with CDK4/6 inhibitors have not been defined. In this study, we evaluated the frequency of ESR1 mutations (both *de novo* and acquired) in cfDNA from patients with MBC undergoing palbociclib and letrozole therapy.

## RESULTS

### Patient clinical characteristics

**Figure 1:**
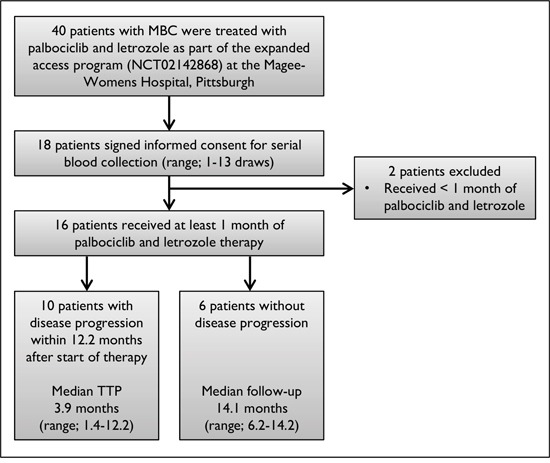
Flow diagram of patient selection Flow chart indicates study population and inclusion criteria for patient selection. TTP represents time to progression.

ESR1 mutations were examined in cfDNA from 16 patients with MBC starting palbociclib and letrozole treatment on an expanded access program (EAP) (NCT02142868, initiated by Pfizer, Inc.). In the EAP, a total of 242 patients with HR+/HER2- advanced breast disease from 42 centers in the US were assigned to single-arm palbociclib 125 mg/d (3 weeks on, 1 week off) in combination with letrozole 2.5 mg/d (continuous daily dosing) until disease progression. Serial blood draws (range; 1-13) were available for 18 out of 40 patients who received treatment at Magee-Womens Hospital, Pittsburgh (Figure 1). The inclusion criterion for our study was that patients received at least one month of palbociclib and letrozole therapy (n=16). No statistical difference in baseline clinical characteristics was observed between patients with wildtype and mutant ESR1, suggesting that the patient population was well-balanced between the comparison groups (Table 1). The median patient age was 63.5 years (range; 39-81), median number of prior therapies was 8 (range; 0-19), and median number of prior endocrine therapies (ET) was 5 (range; 0-9; 2 never with ET, 8 without adjuvant ET). Common prior treatments included anastrozole (81.3%), tamoxifen (75%), fulvestrant (63%), exemestane (50%), and letrozole (31.3%) (Supplementary Table S1). All tumors were positive for ER, 63% positive for PR, and 9.1% positive for HER2. 75% patients had visceral metastases and 68.8% had bone metastases.

**Table 1:** Baseline clinical characteristics in patients with wildtype and mutant ESR1

	ESR1-Wildtype (n=11)	ESR1-Mutant (n=5)	P-value	ESR1-Wildtype (n=11)	ESR1-*De Novo* Mutant (n=3)	P-value
**Age**						
**Median age (yrs)**	65	49	0.36	65	49	0.47
**Range (yrs)**	40-81	39-71		40-81	39-71	
**Race**			0.34			0.34
**White**	10 (90.9%)	5 (100.0%)		10 (90.9%)	3 (100.0%)	
**Asian**	1 (9.1%)	0 (0.0%)		1 (9.1%)	0 (0.0%)	
**Disease Presentation at diagnosis**			0.86			0.76
**Relapsed**	6 (54.5%)	3 (60.0%)		6 (54.5%)	2 (66.7%)	
**Metastatic**	5 (45.5%)	2 (40.0%)		5 (45.5%)	1 (33.3%)	
**Stage (Dx)**			0.49			0.49
**I**	0 (0.0%)	1 (20.0%)		0 (0.0%)	(33.3%)	
**II**	4 (36.4%)	2 (40.0%)		4 (36.4%)	(33.3%)	
**III**	2 (18.2%)	0 (0.0%)		2 (18.2%)	(0.0%)	
**IV**	5 (45.5%)	2 (40.0%)		5 (45.5%)	(33.3%)	
**Hormone-receptor**						
**ER-positive and PR-positive**	7 (63.6%)	3 (60.0%)	0.90	7 (63.6%)	2 (66.7%)	0.94
**ER-positive and HER2-positive**	1 (9.1%)	0 (0.0%)	0.34	1 (9.1%)	(0.0%)	0.34
**Total prior regimens (Chemotherapy + Endocrine)**	8 (range, 0-15)	7 (range, 7-19)	0.34	8 (range, 0-15)	8 (range, 7-19)	0.33
**Adjuvant endocrine regimens**	0 (range, 0-2)	0 (range, 0-2)	0.91	0 (range, 0-2)	0 (range, 0-1)	0.63
**Metastatic endocrine regimens**	2 (range, 0-6)	4 (range, 3-9)	0.13	2 (range, 0-6)	5 (range, 5-9)	0.17
**Visceral metastasis**	8 (72.7%)	4 (80.0%)	0.77	8 (72.7%)	3 (100.0%)	0.08
**Bone metastasis**	7 (63.6%)	4 (80.0%)	0.53	7 (63.6%)	2 (66.7%)	0.94

### ESR1 mutation analysis

**Figure 2:**
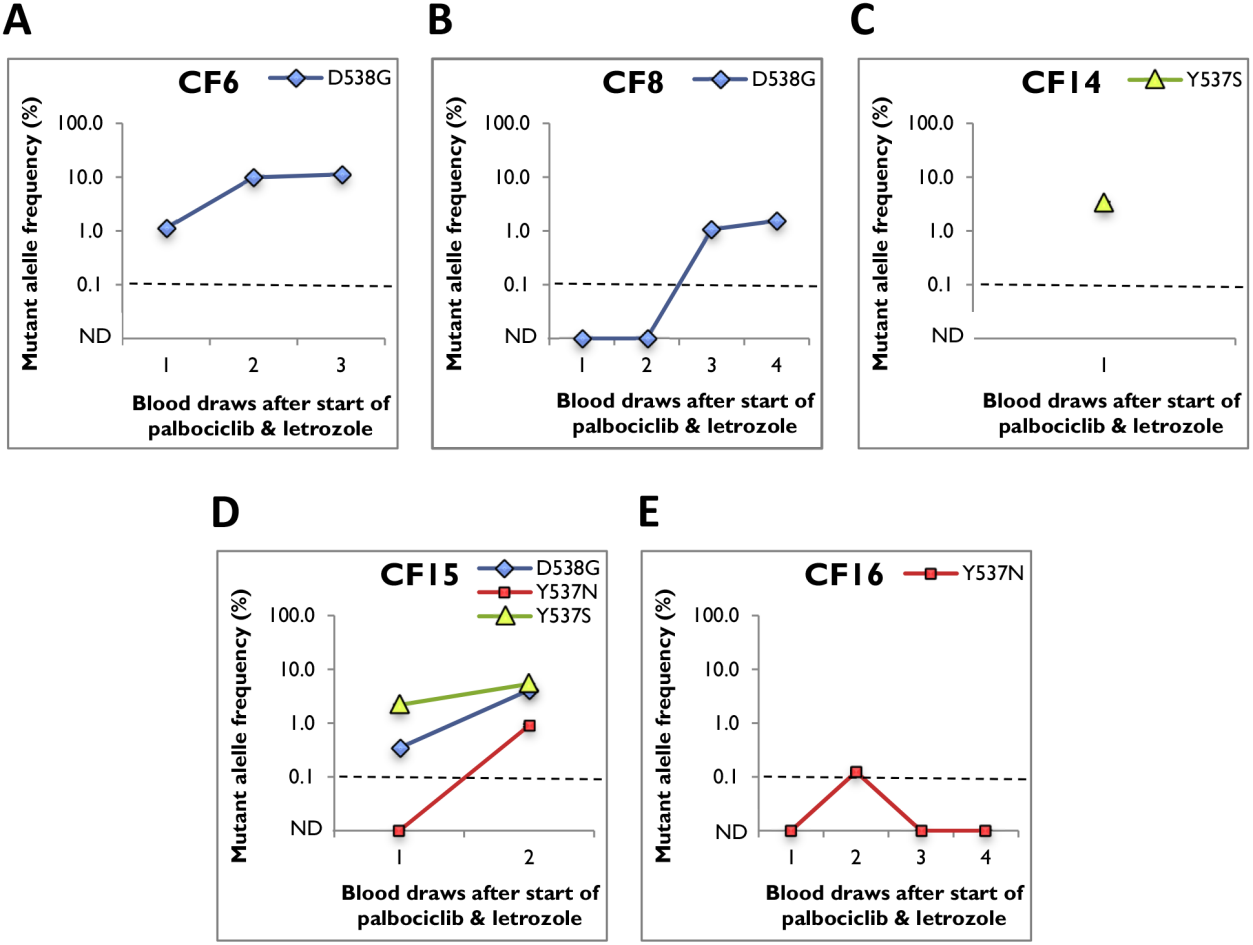
ESR1 mutant allele frequency in serial blood draws from positive cfDNA samples Average mutant allele frequency ± SEM is indicated using data from at least three replicates (after subtraction of background noise). Dotted line representing the LLoD (0.1%) was used as cut-off. ND represents mutation not detected. **A**. patient CF6, **B**. patient CF8, **C**. patient CF14, **D**. patient CF15, and **E**. patient CF16.

**Figure 3:**
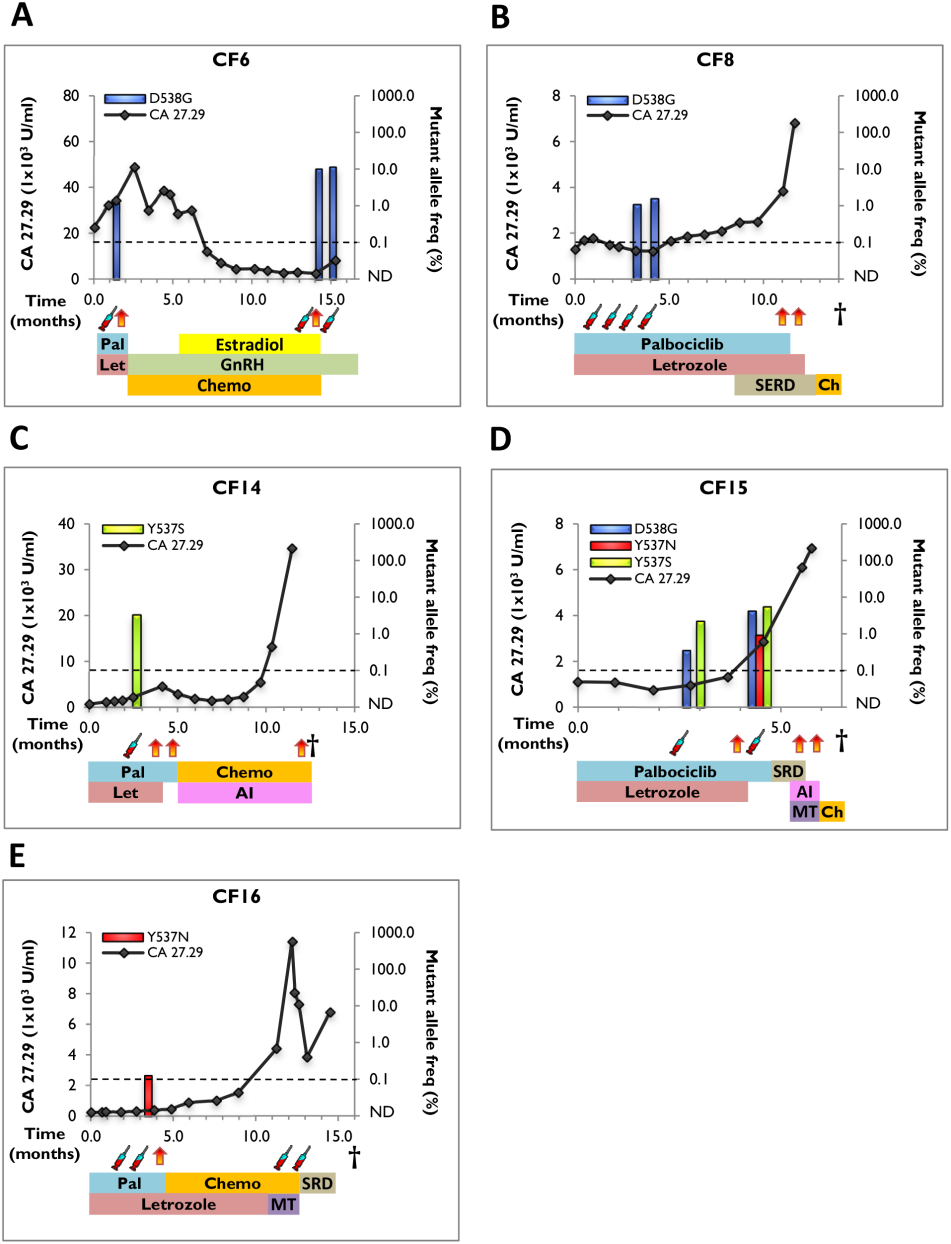
Clinical timeline and mutant allele frequency of ESR1 mutations in serial blood draws from positive cfDNA samples The timeline starts with the initiation of palbociclib and letrozole therapy and shows tumor marker assessments (CA 27.29 antigen line graph), ESR1-mutant allele frequency (bar graphs), LLoD (dotted line), disease progression (orange/red vertical arrows), blood draws (syringe), and treatments received. Treatment abbreviations: Pal (palbociclib), Let (letrozole), SERD (selective estrogen receptor degrader), GnRH (gonadotrophin-releasing hormone), Chemo (chemotherapy), AI (aromatase inhibitor), MT (mTOR inhibitor). **A**. patient CF6, **B**. patient CF8, **C**. patient CF14, **D**. patient CF15, and **E**. patient CF16.

In our previous study, we evaluated six ESR1 mutations (K303R, S463P, Y537C, Y537N, Y537S, and D538G) in primary tumors, metastatic lesions and cfDNA of breast cancer patients [14]. We reported sensitive detection of three ESR1 mutations including D538G, Y537S, and Y537C, but did not detect the Y537N, S463P, and K303R mutations in any of our analyzed samples. Also consistent with recent reports D538G, Y537S, Y537N, and Y537C are the four most frequently detected mutations in ESR1 [10–15]. Hence, in this study we examined these four mutations in serial blood draws from 16 patients with MBC undergoing palbociclib and letrozole therapy. 31.3% (5/16; 95% Wilson binomial CI, 15%-56%) cfDNA samples were positive for ESR1 mutations (n=3, *de novo*, and n=2, acquired) with average allele frequencies ranging from 0.12% to 11.2% (Figure 2, Table 2, and Supplementary Figures S1-S5). Despite low allele frequency (0.12%±0.01), Y537N mutation in patient CF16 was reproducibly positive in multiple repetitive assays and above the LLoD (0.1%) (Figure 2E, Table 2, and Supplementary Figure S5). D538G was the most common mutation (n=3), followed by Y537N and Y537S (n=2 each). Patient CF15 showed polyclonal disease with more than one ESR1 mutation (Figure 2D, Table 2, and Supplementary Figure S4). Longitudinal tracking of ESR1 mutations in serial draws revealed selection of mutations in 3 out of 4 patients while on palbociclib and letrozole therapy, and loss of mutation in 1 patient. Also, the increase in allele frequencies frequently co-occurred with an increase in the tumor marker CA 27.29 (Figure 3).

**Table 2:** ESR1 mutant allele frequency in serial blood draws from positive cfDNA samples

Patient ID	Months After Start of Palbociclib and Letrozole	ESR1-D538G	ESR1-Y537N	ESR1-Y537S
**CF6**				
Draw 1	1.4	1.12 ± 0.04	ND	ND
Draw 2	14.0	9.90 ± 1.18	ND	ND
Draw 3	15.3	11.23 ± 0.29	ND	ND
**CF8**				
Draw 1	1.8	ND	ND	ND
Draw 2	2.3	ND	ND	ND
Draw 3	3.2	1.07 ± 0.08	ND	ND
Draw 4	4.1	1.54 ± 0.10	ND	ND
**CF14**				
Draw 1	2.5	ND	ND	3.26 ± 0.19
**CF15**				
Draw 1	2.8	0.35 ± 0.02	ND	2.18 ± 0.05
Draw 2	4.6	4.12 ± 0.07	0.91 ± 0.05	5.37 ± 0.03
**CF16**				
Draw 1	2.8	ND	ND	ND
Draw 2	3.8	ND	0.12 ± 0.01	ND
Draw 3	13.1	ND	ND	ND
Draw 4	14.5	ND	ND	ND

Average mutant allele frequency ± SEM is indicated using data from at least three replicates (after subtraction of background noise). LLoD of 0.1% was used as cut-off. ND represents mutation not detected.

Patient CF6 presented with stage IV disease with liver metastasis (Table 3 and Supplementary Table S1). She received chemotherapy and ET for 3 years including SERM, AI, SERD, and HER2 inhibitors. Blood drawn after 1.4 months of palbociclib and letrozole treatment was positive for ESR1-D538G mutation (1.1%) (Figure 3A and Table 2). Due to disease progression at the time of first blood draw, treatment was switched to chemotherapy, gonadotrophin-releasing hormone (GnRH), and estradiol. Subsequent blood draws after a year showed enrichment of the mutation (9.9% and 11.2%), which also corresponded with increased CA 27.29 levels.

**Table 3:** Clinical characteristics and treatment history in patients with ESR1 mutation

Mutations and Clinical Characteristics					Treatment History						
					Therapy Prior to Start of Palbociclib and Letrozole		Therapy Prior to 1^st^ Mutation Analysis		Therapy Post 1^st^ Mutation Analysis		Total Palbociclib and Letrozole
Patient ID	Detected ESR1 Mutations	*De Novo* Vs Acquired	Stage at Dx	ER Status	Therapy Type	Cumulative Exposure (months)	Therapy Type	Cumulative Exposure (months)	Therapy Type	Cumulative Exposure (months)	Cumulative Exposure (months)
CF6	D538G	*De Novo*	IV	+	SERM, AI, SERD, HER2i, Chemo	36.0	Palbociclib, Letrozole	1.4	Estradiol, GnRH, Chemo	14.3	1.4
CF8	D538G	Acq.	IV	+	SERM, AI, SERD, Chemo	36.0	Palbociclib, Letrozole	2.0	Palbociclib, Letrozole, SERD, Chemo	10.1	12.1
CF14	Y537S	*De Novo*	IIB	+	SERM, AI, SERD, Chemo	93.0	Palbociclib, Letrozole	2.5	Palbociclib, Letrozole, Chemo, AI	9.4	4.2
CF15	D538G, Y537N, Y537S	*De Novo*	IA	+	SERM, AI, SERD, mTORi, Chemo	24.0	Palbociclib, Letrozole	3.0	Palbociclib, Letrozole, SERD, AI, mTORi, Chemo	3.8	4.8
CF16	Y537N	Acq.	IIA	+	SERM, AI, SERD, Chemo	12.0	Palbociclib, Letrozole	3.0	Palbociclib, Letrozole, Chemo, mTORi, SERD	11.4	11.3

For patient CF8, four serial blood draws were obtained. The patient was diagnosed with stage IV disease with bone metastasis, and received serial ET (SERM, AI, and SERD) and chemotherapy for 3 years before starting palbociclib and letrozole (Table 3 and Supplementary Table S1). First blood sample was collected 1.8 months after exposure to palbociclib and letrozole, followed by three additional monthly draws (Figure 3B and Table 2). The patient acquired ESR1-D538G mutation in the third draw (1.1%) with an increase in allele frequency to 1.5% in the fourth draw, which coincided with a decrease in CA 27.29 levels. Disease progression was seen 8 months after the fourth draw, and therapy was switched to SERD and chemotherapy for 3 months before death.

Patient CF14 developed bone metastasis 11 years after the diagnosis of ER+/PR+ primary breast cancer (Supplementary Table S1). Prior to palbociclib and letrozole, she received extensive ET (SERM, AI, and SERD), both in the adjuvant and metastatic setting (Table 3 and Supplementary Table S1). cfDNA analysis revealed ESR1-Y537S mutation (3.3%) in blood collected 2.5 months after exposure to palbociclib and letrozole (Figure 3C and Table 2). The disease progressed and she received additional AI and chemotherapy in the last 6 months before death.

Patient CF15 developed metastases to liver and bone 4 years after the diagnosis of invasive ductal carcinoma (IDC) (Supplementary Table S1). She received SERM, SERD, AI, mTOR inhibitor, and chemotherapy for 2 years before starting palbociclib and letrozole. A blood draw after 2.8 months showed two ESR1 mutations (D538G - 0.35% and Y537S - 2.2%) (Figure 3D and Table 2). Interestingly, in the subsequent draw 7.6 months after initiation of palbociclib and letrozole, there was enrichment of the two ESR1 mutations (D538G – 4.1% and Y537S – 5.4%) and appearance of a third ESR1 mutation, Y537N (0.91%). Increasing mutant allele frequencies co-occurred with increase in CA 27.29 tumor marker. Disease progressed and patient received SERD, AI, mTOR inhibitor and chemotherapy within 2 months before death.

Patient CF16 developed multiple metastases to liver, bone, and brain 7 years after the diagnosis of primary tumor (Supplementary Table S1). She received SERM, SERD, AI, and chemotherapy for a year before starting palbociclib and letrozole. Four blood draws were obtained at months 3, 4, 13, and 14 after the initiation of palbociclib and letrozole (Figure 3E and Table 2). A low frequency ESR1-Y537N mutation was acquired in the second draw (0.12%), which corresponded to an increase in CA 27.29. However, the mutation was below LLoD in subsequent draws.

### Progression-free survival and overall survival

**Figure 4:**
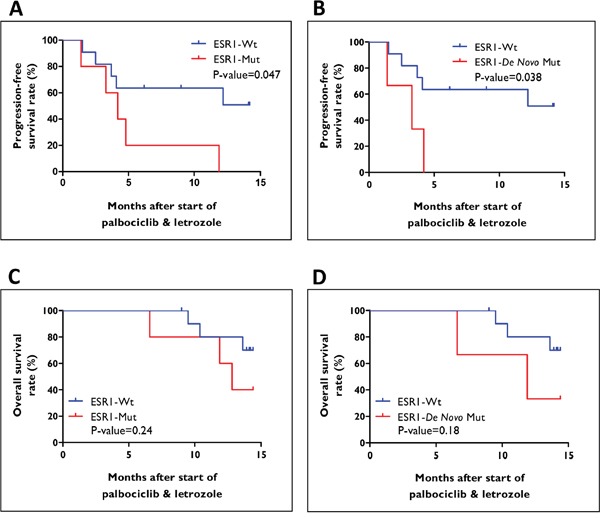
Survival curves for patients treated with palbociclib and letrozole **A-B**. Progression-free survival (PFS), and **C-D**. overall survival (OS) for patients stratified based on ESR1 mutation status, ESR1-wildtype versus ESR1-mutant, and ESR1-wildtype versus ESR1-*de novo* mutant.

We next examined the impact of ESR1 mutations on clinical outcome in the 16 patients comparing ESR1-wildtype and -mutant groups for differences in PFS and OS (Figure 4). PFS in ESR1-wildtype group (n=11) ranged from 1.6 months to 14.2 months, with a median value of 9.0 months. On the other hand, patients where ESR1 mutation was detected at any given time during treatment (i.e. ESR1-mutant group; n=5), PFS ranged from 1.4 months to 12.0 months, with a median value of 4.2 months. Hence, faster progression was seen in the ESR1-mutant group, but this only approached significance (HR=0.31; 95% CI: 0.05-0.95, log-rank P-value=0.047). However, PFS was significantly shorter in patients already carrying ESR1 mutation at initial blood draw (i.e. *ESR1-de novo* mutant group; n=3) (median; 9.0 versus 3.3 months, HR=0.25; 95% CI: 0.01-0.9, P-value=0.038).

No significant difference in OS (median; 14.1 versus 12.8 months, HR=0.40; 95% CI: 0.06-2.0, P-value=0.24) was observed between patients with wildtype and mutant ESR1. Furthermore, median OS in the ESR1-*de novo* mutant group was 12.0 months with a trend towards significance for faster progression (HR=0.32; 95% CI: 0.02-2.1, P-value=0.18).

PFS (9.0 versus 8.4 months, HR=0.36; 95% CI: 0.05-2.9, P-value=0.34) and OS (14.1 versus 13.6 months, HR=0.54; 95% CI: 0.04-7.6, P-value=0.65) were similar in patients with wildtype ESR1 and patients who acquired ESR1 mutation during palbociclib and letrozole therapy.

## DISCUSSION

There is growing recognition that ESR1 mutations are relatively uncommon in newly diagnosed, treatment-naive breast cancer, but frequently acquired in hormone-resistant metastatic breast cancer. CDK4/6 inhibition in combination with endocrine therapy is a promising new therapeutic strategy in hormone receptor-positive advanced breast cancer. However, the incidence and impact of ESR1 mutations on clinical outcome in patients treated with CDK4/6 inhibitors have not been defined. This is the first study to evaluate the frequency of ESR1 mutations (D538G, Y537C, Y537N, and Y537S) in cfDNA from patients with MBC treated with palbociclib and letrozole using ddPCR. We found that 31.3% (5/16) cfDNA samples were positive for ESR1 mutations (n=3, *de novo*, and n=2, acquired) with average allele frequencies ranging from 0.12% to 11.2%. The ESR1 mutation prevalence in cfDNA is similar to our previous report (24.1%) from patients with advanced hormone-refractory breast cancer. Also, consistent with our previous report, D538G was the most common ESR1 mutation (n=3), followed by Y537S (n=2). However, we did not detect any Y537C mutation in this patient cohort, but instead Y537N mutation (n=2). Further, cfDNA analysis in patient CF15 showed three different ESR1 mutations (D538G, Y537S, and Y537N). D538G (0.35%) and Y537S (2.2%) mutations were detected in the initial blood draw, 2.8 months after the initiation of palbociclib and letrozole treatment. We observed an increase in the mutant allele frequencies in subsequent draw (D538G – 4.1% and Y537S – 5.4%) and appearance of Y537N (0.91%). The co-occurrence of multiple ESR1 mutations and enrichment on palbociclib and letrozole therapy suggests a role for selection pressure during drug treatment and convergent evolution.

cfDNA analysis can facilitate longitudinal monitoring of ESR1 mutations and association with response to endocrine therapy. In our study, longitudinal assessment of ESR1 mutations in serial blood draws revealed selection of mutations in 3 out of 4 patients while on palbociclib and letrozole therapy, and loss of mutation in 1 patient. The combination of palbociclib and AI did not prevent the selection of ESR1 mutations when used in later lines of therapy. Also, the increase in mutant allele frequencies frequently co-occurred with an increase in the tumor marker CA 27.29. These results suggest that ESR1 mutations may be predictive of development of resistance during palbociclib and AI therapy. However, our results are limited by the small number of blood draws that were available for patients with ESR1 mutations (range; 1-4), unlike frequent measurements of the tumor marker CA 27.29. Hence, larger studies utilizing more frequent analysis of cfDNA are needed to further validate these findings. An additional limitation of our data is that the patient population was heterogeneous and heavily pretreated; therefore these results may be less applicable in the currently approved treatment setting of palbociclib, which is in 1st and 2nd line relatively treatment-naïve patients.

We examined the association between ESR1 mutation status and clinical outcome in patients with MBC treated with palbociclib and letrozole. PFS (4.2 versus 9.0 months, P-value=0.047) and OS (12.8 versus 14.1 months, P-value=0.24) were similar in patients with and without ESR1 mutation detected at any point in time during treatment. However, PFS was significantly shorter in patients already carrying ESR1 mutation at the time of first blood draw after initiation of palbociclib and letrozole therapy (i.e ESR1-*de novo* mutant group) (3.3 versus 9.0 months, P-value=0.038). Despite the interesting finding, this analysis is limited by the small sample size (ESR1-*de novo* mutant group; n=3, ESR1-wildtype group; n=11) and validation in larger clinical trials is warranted. Another limitation of our study is the absence of a control arm (letrozole plus placebo group). Hence, the clinical relevance of this association between ESR1 mutation status and response to palbociclib and letrozole therapy cannot be fully addressed. A recent abstract submitted to the American Society of Clinical Oncology (ASCO) on the PALOMA-3 trial showed that the combination of palbociclib and fulvestrant provided significant clinical benefit in patients both with and without ESR1 mutations [23]. It is notable, however, that fulvestrant has not been associated with development of ESR1 mutations unlike the AI's and may be a viable treatment strategy for patients with ESR1 mutations. More studies that assess ESR1 mutations longitudinally in patients treated with palbociclib will be required to evaluate the efficacy of CDK4/6 inhibitors as a novel targeted therapy that may overcome this specific mechanism of endocrine resistance.

In summary, sensitive detection of ESR1 mutations in patients with MBC treated with palbociclib and letrozole reveal 31.3% mutation rate, which is consistent with recent reports. 6.3% patients showed multiple ESR1 mutations. Mutations were enriched in serial blood draws, suggesting that treatment with palbociclib and letrozole does not prevent the selection of ESR1 mutations in later lines of therapy. PFS and OS were similar in patients with and without mutation detected at any given time during treatment. However, PFS was significantly shorter in patients already carrying ESR1 mutation at initial blood draw.

## MATERIALS AND METHODS

### Collection of blood samples

20ml venous blood was collected in Streck Cell-free DNA blood tubes from 16 patients with MBC with signed informed consent under the University of Pittsburgh IRB approved protocol (IRB0502025). Serial blood draws (range; 1-13) were obtained from the University of Pittsburgh Health Sciences Tissue Bank (HSTB). A total of 60 samples from 16 patients were processed for cfDNA isolation and mutation testing.

### cfDNA isolation and quantification

cfDNA was isolated as previously described [14]. Blood plasma was separated by double centrifugation within 4 days of blood collection. 1 to 4ml of plasma was used for isolation of cfDNA using QIAamp Circulating Nucleic Acid kit (Qiagen). cfDNA was quantified using Qubit dsDNA HS assay kit (Life Technologies).

### Targeted preamplification

We have previously shown that targeted preamplification enables generation of sufficient quantities of cfDNA for use in ddPCR, but does not affect the linearity of mutant allele detection [14]. 2 ng of cfDNA was subjected to targeted high-fidelity preamplification for 15 cycles using primers (Supplementary Table S2) and PCR conditions previously described. Preamplification products were purified using QIAquick PCR Purification kit (Qiagen) and diluted at 1:20 before use in ddPCR reaction.

### Droplet digital PCR

Bio-Rad QX100 droplet digital PCR platform was used for sensitive detection of ESR1 mutations. Primers and probes were ordered for D538G (Integrated DNA Technologies) and Y537C/N/S (Life Technologies) mutations using sequences (Supplementary Table S3) previously described [14]. 1ul of diluted preamplified cfDNA was used as input for ddPCR reaction. Water and ESR1 wildtype DNA as negative controls, and oligonucleotides carrying mutation of interest or DNA from a cell line with knock-in mutation as positive controls were included in each run to eliminate potential false-positive mutant signals. All mutation-positive samples were run in triplicates, assaying at least 10,000 genome equivalents.

Data were analyzed using Bio-Rad QuantaSoft package. Mutant allele frequency was calculated as mutant concentration relative to total concentration of mutant plus wildtype in copies/ul. Background noise was defined as the average of allele frequency plus half 95% confidence intervals of negative controls (ESR1 wildtype DNA) across all ddPCR assays. The noise (if any) was subtracted from the allele frequencies. The background noise-adjusted 0.1% lower limit of detection (LLoD) previously described for cfDNA was used as cut-off [14].

### Survival analysis

PFS and OS were assessed from the start of palbociclib and letrozole therapy to the date of disease progression and date of death respectively. Disease progression was determined by rising serum CA 27-29 and progression on imaging of metastatic area (IE - CT, PET) or death; on two occasions, progression determined by biopsy result or severe clinical symptoms (RUQ pain, nausea) in setting of rising CA 27-29 (not attributed to toxicity of palbociclib or letrozole toxicity). End-date for the study, April 11^th^, 2016 was used for the survival analysis. Log-rank test stratified according to the presence or absence of events of progression or death was used to compare PFS and OS respectively, between the ESR1-wildtype and -mutant groups. All reported P values were two-sided.

## SUPPLEMENTARY FIGURES AND TABLES





## References

[1] (1998). Tamoxifen for early breast cancer: an overview of the randomised trials. Early Breast Cancer Trialists' Collaborative Group. Lancet.

[2] Dowsett M, Cuzick J, Ingle J, Coates A, Forbes J, Bliss J, Buyse M, Baum M, Buzdar A, Colleoni M, Coombes C, Snowdon C, Gnant M, Jakesz R, Kaufmann M, Boccardo F (2010). Meta-analysis of breast cancer outcomes in adjuvant trials of aromatase inhibitors versus tamoxifen. J Clin Oncol.

[3] Sainsbury R (2013). The development of endocrine therapy for women with breast cancer. Cancer Treat Rev.

[4] Sini V, Cinieri S, Conte P, De Laurentiis M, Leo AD, Tondini C, Marchetti P (2016). Endocrine therapy in post-menopausal women with metastatic breast cancer: From literature and guidelines to clinical practice. Crit Rev Oncol Hematol.

[5] Andre F, Slimane K, Bachelot T, Dunant A, Namer M, Barrelier A, Kabbaj O, Spano JP, Marsiglia H, Rouzier R, Delaloge S, Spielmann M (2004). Breast cancer with synchronous metastases: trends in survival during a 14-year period. J Clin Oncol.

[6] Musgrove EA, Sutherland RL (2009). Biological determinants of endocrine resistance in breast cancer. Nat Rev Cancer.

[7] Osborne CK, Schiff R (2011). Mechanisms of endocrine resistance in breast cancer. Annu Rev Med.

[8] Stephens PJ, Tarpey PS, Davies H, Van Loo P, Greenman C, Wedge DC, Nik-Zainal S, Martin S, Varela I, Bignell GR, Yates LR, Papaemmanuil E, Beare D, Butler A, Cheverton A, Gamble J (2012). The landscape of cancer genes and mutational processes in breast cancer. Nature.

[9] Curtis C, Shah SP, Chin SF, Turashvili G, Rueda OM, Dunning MJ, Speed D, Lynch AG, Samarajiwa S, Yuan Y, Graf S, Ha G, Haffari G, Bashashati A, Russell R, McKinney S (2012). The genomic and transcriptomic architecture of 2,000 breast tumours reveals novel subgroups. Nature.

[10] Robinson DR, Wu YM, Vats P, Su F, Lonigro RJ, Cao X, Kalyana-Sundaram S, Wang R, Ning Y, Hodges L, Gursky A, Siddiqui J, Tomlins SA, Roychowdhury S, Pienta KJ, Kim SY (2013). Activating ESR1 mutations in hormone-resistant metastatic breast cancer. Nat Genet.

[11] Toy W, Shen Y, Won H, Green B, Sakr RA, Will M, Li Z, Gala K, Fanning S, King TA, Hudis C, Chen D, Taran T, Hortobagyi G, Greene G, Berger M (2013). ESR1 ligand-binding domain mutations in hormone-resistant breast cancer. Nat Genet.

[12] Merenbakh-Lamin K, Ben-Baruch N, Yeheskel A, Dvir A, Soussan-Gutman L, Jeselsohn R, Yelensky R, Brown M, Miller VA, Sarid D, Rizel S, Klein B, Rubinek T, Wolf I (2013). D538G mutation in estrogen receptor-alpha: A novel mechanism for acquired endocrine resistance in breast cancer. Cancer Res.

[13] Jeselsohn R, Yelensky R, Buchwalter G, Frampton G, Meric-Bernstam F, Gonzalez-Angulo AM, Ferrer-Lozano J, Perez-Fidalgo JA, Cristofanilli M, Gomez H, Arteaga CL, Giltnane J, Balko JM, Cronin MT, Jarosz M, Sun J (2014). Emergence of constitutively active estrogen receptor-alpha mutations in pretreated advanced estrogen receptor-positive breast cancer. Clinical cancer research.

[14] Wang P, Bahreini A, Gyanchandani R, Lucas PC, Hartmaier RJ, Watters RJ, Jonnalagadda AR, Trejo Bittar HE, Berg A, Hamilton RL, Kurland BF, Weiss KR, Mathew A, Leone JP, Davidson NE, Nikiforova MN (2016). Sensitive Detection of Mono- and Polyclonal ESR1 Mutations in Primary Tumors, Metastatic Lesions, and Cell-Free DNA of Breast Cancer Patients. Clinical cancer research.

[15] Chu D, Paoletti C, Gersch C, VanDenBerg DA, Zabransky DJ, Cochran RL, Wong HY, Toro PV, Cidado J, Croessmann S, Erlanger B, Cravero K, Kyker-Snowman K, Button B, Parsons HA, Dalton WB (2016). ESR1 Mutations in Circulating Plasma Tumor DNA from Metastatic Breast Cancer Patients. Clinical cancer research.

[16] Musgrove EA, Caldon CE, Barraclough J, Stone A, Sutherland RL (2011). Cyclin D as a therapeutic target in cancer. Nat Rev Cancer.

[17] Lundgren K, Brown M, Pineda S, Cuzick J, Salter J, Zabaglo L, Howell A, Dowsett M, Landberg G (2012). Effects of cyclin D1 gene amplification and protein expression on time to recurrence in postmenopausal breast cancer patients treated with anastrozole or tamoxifen: a TransATAC study. Breast Cancer Res.

[18] Thangavel C, Dean JL, Ertel A, Knudsen KE, Aldaz CM, Witkiewicz AK, Clarke R, Knudsen ES (2011). Therapeutically activating RB: reestablishing cell cycle control in endocrine therapy-resistant breast cancer. Endocr Relat Cancer.

[19] Dhillon S (2015). Palbociclib: first global approval. Drugs.

[20] Finn RS, Crown JP, Lang I, Boer K, Bondarenko IM, Kulyk SO, Ettl J, Patel R, Pinter T, Schmidt M, Shparyk Y, Thummala AR, Voytko NL, Fowst C, Huang X, Kim ST (2015). The cyclin-dependent kinase 4/6 inhibitor palbociclib in combination with letrozole versus letrozole alone as first-line treatment of oestrogen receptor-positive, HER2-negative, advanced breast cancer (PALOMA-1/TRIO-18): a randomised phase 2 study. Lancet Oncol.

[21] Turner NC, Ro J, Andre F, Loi S, Verma S, Iwata H, Harbeck N, Loibl S, Huang Bartlett C, Zhang K, Giorgetti C, Randolph S, Koehler M, Cristofanilli M (2015). Palbociclib in Hormone-Receptor-Positive Advanced Breast Cancer. N Engl J Med.

[22] Schiavon G, Hrebien S, Garcia-Murillas I, Cutts RJ, Pearson A, Tarazona N, Fenwick K, Kozarewa I, Lopez-Knowles E, Ribas R, Nerurkar A, Osin P, Chandarlapaty S, Martin LA, Dowsett M, Smith IE (2015). Analysis of ESR1 mutation in circulating tumor DNA demonstrates evolution during therapy for metastatic breast cancer. Sci Transl Med.

[23] Turner NC, Jiang Y, O'Leary B, Hrebien S, Cristofanilli M, Andre F, Loibl S, English PA, Zhang K, Randolph S, Bartlett CH, Koehler M, Loi S (2016). Efficacy of palbociclib plus fulvestrant (P+F) in patients (pts) with metastatic breast cancer (MBC) and ESR1 mutations (mus) in circulating tumor DNA (ctDNA). J Clin Oncol.

