# Supplementary material for: Detection of ESR1 mutations in circulating cell-free DNA from patients with metastatic breast cancer treated with palbociclib and letrozole
Source: Oncotarget. 2016 Aug 19;8(40):66901–11. doi: 10.18632/oncotarget.11383 (PMC5620144; doi:10.18632/oncotarget.11383)
Supplement: Supplementary file 1 [file oncotarget-08-66901-s001.pdf]

Detection of ESR1 mutations in circulating cell-free DNA from patients with metastatic breast cancer treated with palbociclib and letrozole

SUPPLEMENTARY FIGURES AND TABLES

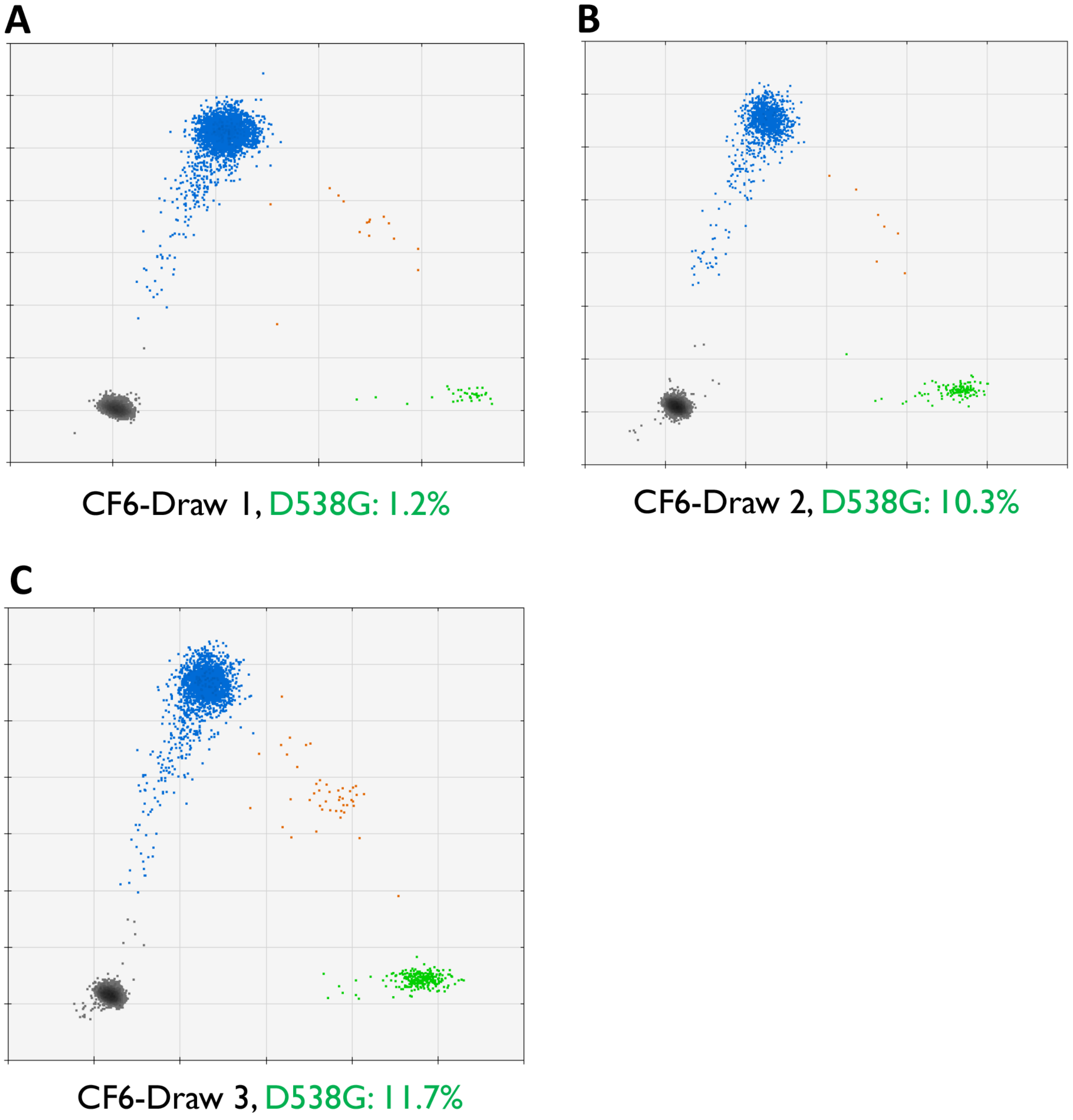

Supplementary Figure S1: ESR1-D538G mutant allele frequency in serial blood draws is indicated for patient CF6 using data from a representative experiment (after subtraction of background noise).

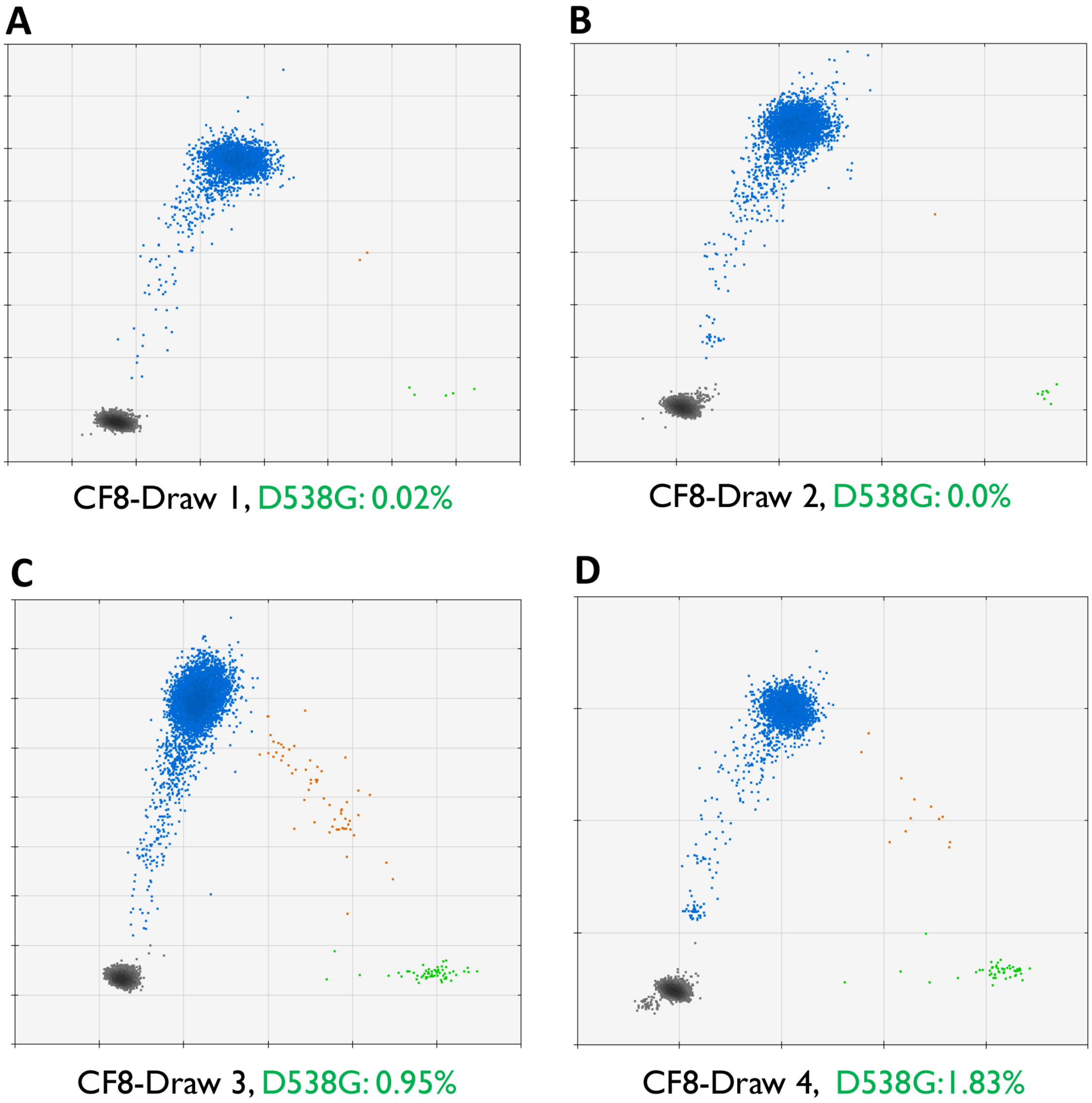

Supplementary Figure S2: ESR1-D538G mutant allele frequency in serial blood draws is indicated for patient CF8 using data from a representative experiment (after subtraction of background noise).

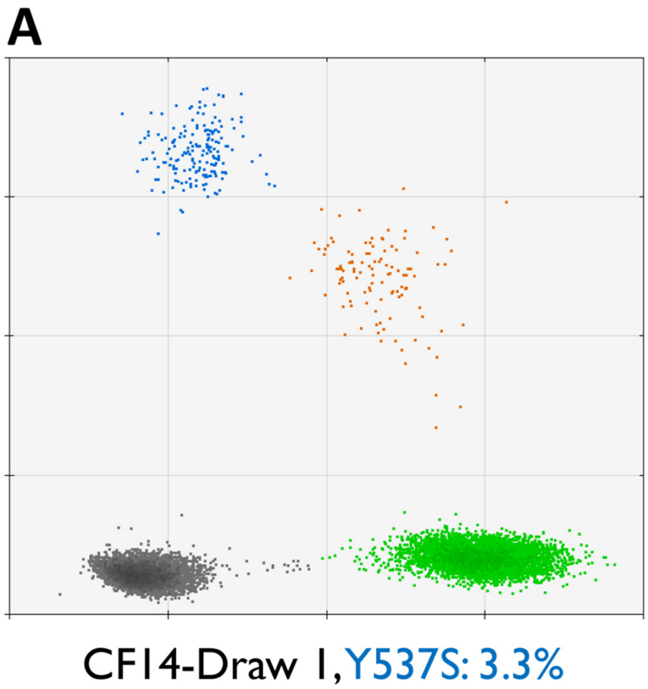

Supplementary Figure S3: ESR1-Y537S mutant allele frequency is indicated for patient CF14 using data from a representative experiment (after subtraction of background noise).

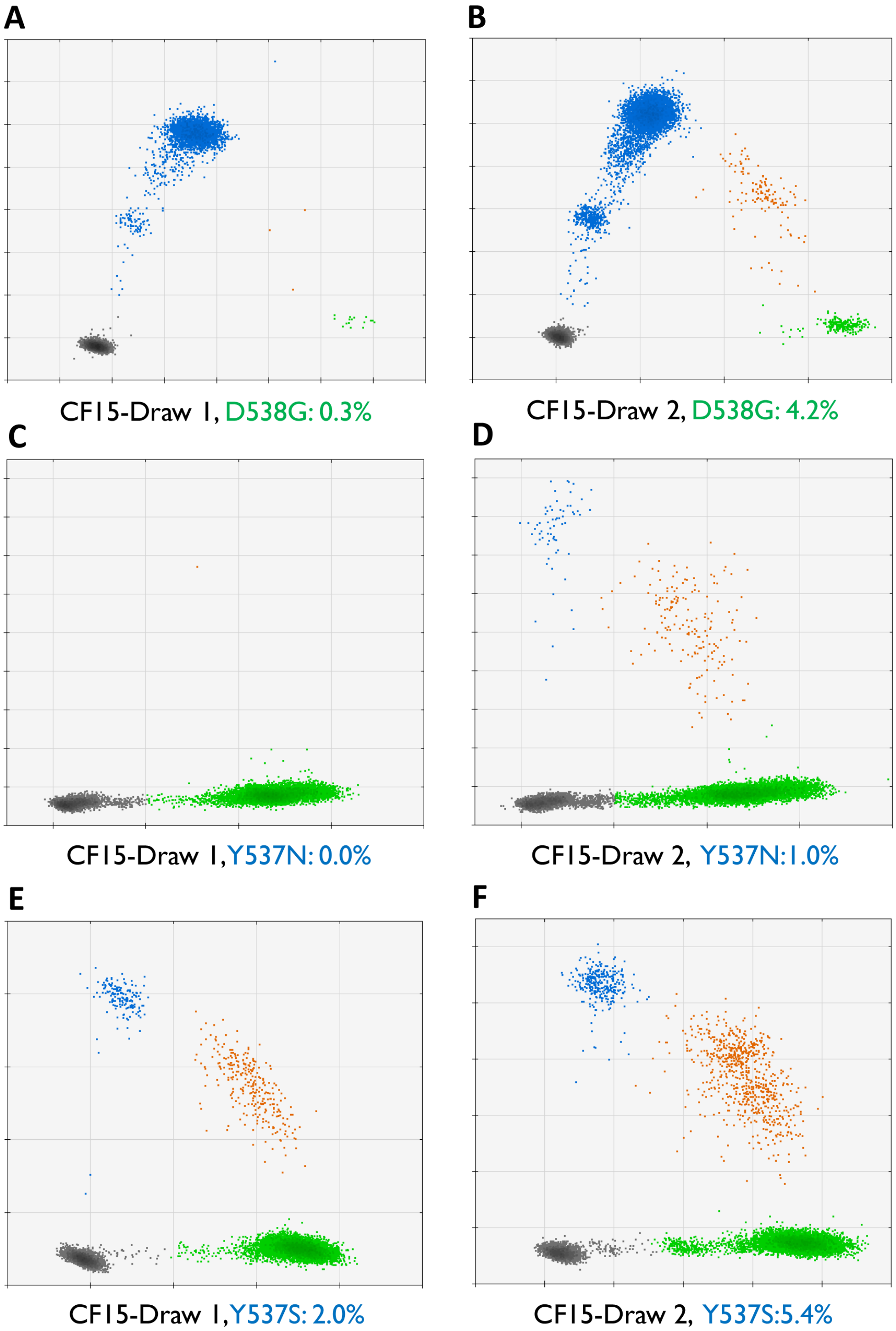

Supplementary Figure S4: ESR1-D538G, -Y537N, and -Y537S mutant allele frequencies in serial blood draws are indicated for patient CF15 using data from a representative experiment (after subtraction of background noise).

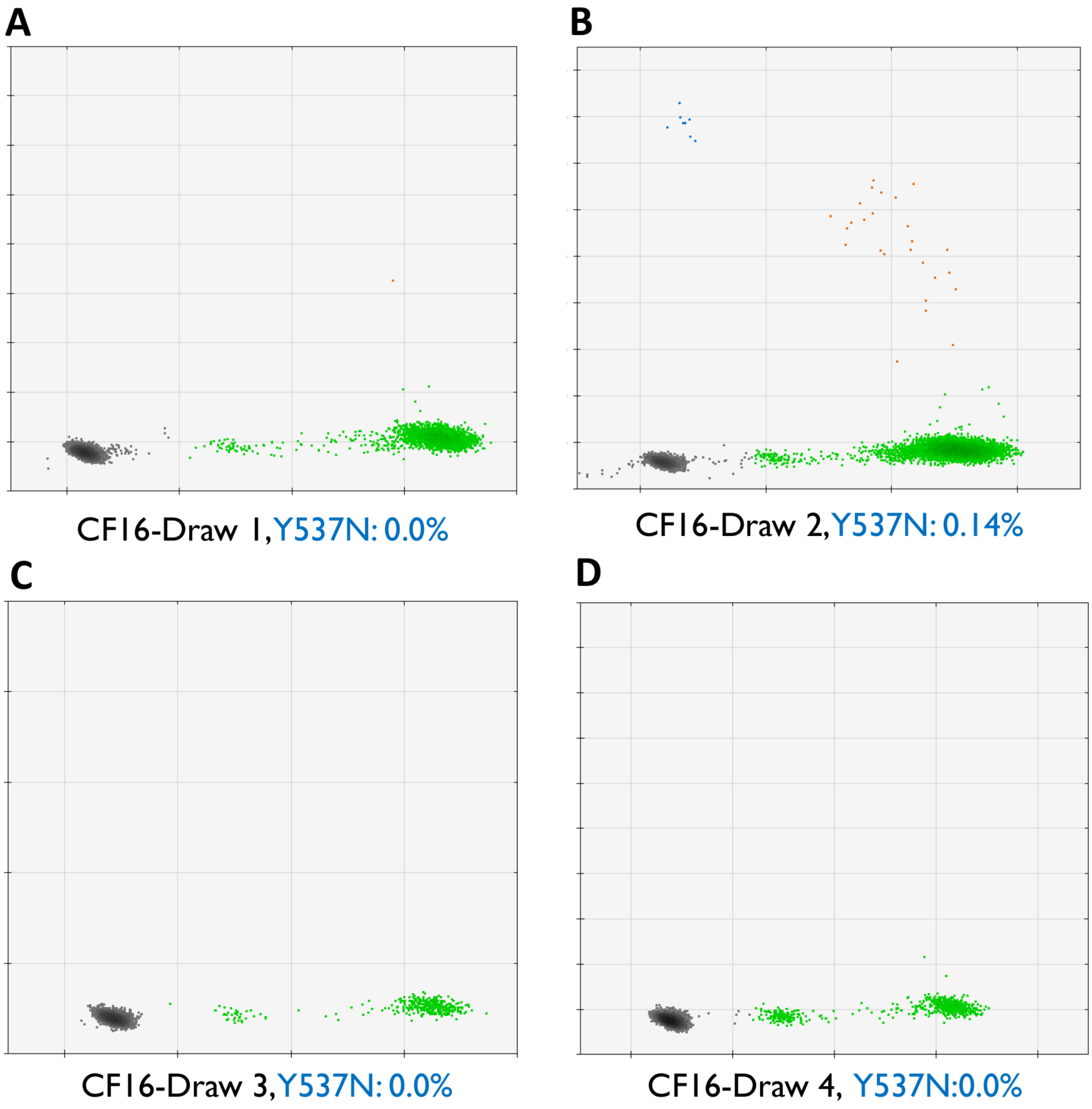

Supplementary Figure S5: ESR1-Y537N mutant allele frequency in serial blood draws is indicated for patient CF16 using data from a representative experiment (after subtraction of background noise).

Supplementary Table S1: Clinical characteristics and treatment data for all patients

See Supplementary File 1

Supplementary Table S2: Sequence of targeted amplification primers

Mutations	Forward primer	Reverse primer	Amplicon size
ESR1 Y537C *ESR1* Y537N *ESR1* Y537S *ESR1* D538G	CAAAGGCATGGAGCATCTGTA	TGAAGTAGAGCCCGCAGT	169

Supplementary Table S3: Sequence of ddPCR primer and probes

Mutation	Forward primer	Reverse primer	Mutant Probe	WT probe	Fluorescence
ESR1-Y537C	CAGCATGAAGTGC AAGAACGT	TGGGCGTCCAGCA TCTC	CCCCTCTGTG ACCTG	TGCCCCTCTAT GACCTG	FAM/VIC
ESR1-Y537N	CTGTACAGCATGA AGTGCAAGAAC	TGGGCGTCCAGCA TCTC	TGCCCCTC AATGAC	TGGTGCCCCT CTATGAC	FAM/VIC
ESR1-Y537S	CAGCATGAAGTGC AAGAACGT	TGGGCGTCCAGCA TCTC	CCCTCTCTG ACCTGC	CCCCTCTAT GACCTGC	FAM/VIC
ESR1-D538G	GCATGAAGTGCAA GAACGTG	AAGTGGCTTTGGTC CGTCT	TCTATGGCCTGCTGCT GGAGATGCT	TCTATGACCTGCT GCTGGAGATGCT	HEX/FAM
